# Special Issue “Molecular Perspectives in Lung Diseases: Pathogenesis, Diagnosis, and Treatment”

**DOI:** 10.3390/ijms27114652

**Published:** 2026-05-22

**Authors:** Elena Levantini

**Affiliations:** 1 CNR-Consiglio Nazionale delle Ricerche, Istituto di Tecnologie Biomediche (ITB-CNR), Area della Ricerca di Pisa, 56124 Pisa, PI, Italy; elena.levantini@itb.cnr.it; 2 Fondazione Pisana per la Scienza ONLUS (FPS), 56017 San Giuliano Terme, PI, Italy

The molecular understanding of lung diseases is rapidly evolving, driven by the convergence of diagnostic innovation, mechanistic insights, and therapeutic exploration. The twelve contributions included in this Special Issue (see Table 1) highlight how diverse pathological processes, from chronic inflammation to tumor progression, are increasingly understood through interconnected molecular programs that bridge immune regulation, cellular plasticity, and systemic responses (see Figure 1).

A central theme emerging from this collection is the growing importance of non-invasive molecular biomarkers for early detection and disease stratification. Several contributions emphasize how circulating mediators reflect the dynamic interplay between tumor cells, immune surveillance, and systemic inflammation. In particular, the identification of interleukin-based signatures capable of distinguishing early-stage non-small cell lung cancer (NSCLC) from both healthy individuals and inflammatory conditions underscores the diagnostic value of immune-derived signals (Morjani et al.). Their study is especially notable for the development of a multi-cytokine diagnostic model integrating IL-6, IL-10, IL-8, and IL-1RA, which achieved superior discriminatory performance compared to individual biomarkers alone. By combining inflammatory mediators associated with tumor-promoting signaling, immune modulation, and chronic inflammatory responses, this composite plasma signature highlights how integrated immune profiling may improve the sensitivity and specificity of early NSCLC detection, representing a promising yet still evolving diagnostic strategy, particularly in clinically challenging settings such as differentiation from chronic obstructive pulmonary disease (COPD).

Complementary approaches based on circulating nucleic acids further reinforce this paradigm, with microRNA panels providing robust and biologically relevant candidates for early detection through liquid biopsy strategies (Zedan et al.). Beyond descriptive cataloging, this work integrates biological relevance with methodological and translational considerations. The study systematically examines miRNAs involved in major oncogenic pathways, including EMT, PI3K/AKT/mTOR, and JAK-STAT signaling, while also critically addressing key technical challenges related to quantification, normalization, and reproducibility in circulating miRNA analyses. By ranking the most clinically and analytically promising candidates and discussing optimized normalization strategies, the authors establish a robust foundation for the development of standardized miRNA-based diagnostic panels for early-stage NSCLC detection.

Similarly, transcriptomic profiling of peripheral blood mononuclear cells (PBMCs) reveals gene signatures linked to COPD, highlighting the potential of accessible systemic biomarkers to capture early disease alterations (Savin et al.). A key strength of this study lies in its integration of systems biology with clinical validation through an analytical strategy combining Weighted Gene Co-expression Network Analysis (WGCNA) with protein–protein interaction mapping, independent dataset validation, and experimental confirmation in patient-derived samples. This approach enabled the identification of a robust PBMC-associated gene signature, including MDM2, FKBP5, and CTNNA1, linked to immune regulation and tissue remodeling pathways, further supporting the concept that peripheral blood profiling may provide a minimally invasive window into early COPD pathogenesis.

Beyond detection, the contributions in this Special Issue collectively emphasize the central role of inflammation and immune modulation across lung pathologies, providing a unifying conceptual thread across diverse disease contexts. Cytokine networks, epithelial responses, and innate immune pathways emerge as key regulators of disease progression. Crucially, heightened inflammatory signaling and impaired antiviral responses contribute to increased susceptibility to viral infections in COPD, pointing to host–pathogen interactions as important contributors to disease exacerbation (Bai H et al.). Using a human lung airway-on-a-chip system that recapitulates key features of the airway microenvironment, the authors demonstrate increased viral susceptibility of COPD-derived epithelium, reflecting an intrinsically dysregulated epithelial state. This model reveals a shift towards a pro-inflammatory and virus-permissive phenotype with impaired barrier integrity and enhanced mucus production. At the transcriptional level, COPD epithelial cells display upregulation of serine proteases together with suppression of interferon-mediated antiviral pathways, indicating combined defects in barrier defense and innate immunity. The ability of the protease inhibitor nafamostat to partially reverse these alterations supports the development of targeted strategies for influenza-associated COPD exacerbations.

Chronic inflammatory programs, such as those driven by TGF-β (Sanchez-Rojas et al.), further demonstrate context-dependent effects, promoting tumor progression under specific conditions. Importantly, this work integrates mechanistic insights from a comorbidity model with clinically relevant observations in patient samples, linking airway inflammation to metastatic dissemination. Through this combined approach, the authors show increased pulmonary metastatic burden associated with enhanced TGF-β expression and proliferative signaling, while the correlation of TGF-β and PCNA expression in patient specimens and their association with poor overall survival further supports the clinical relevance of these findings. This integrative evidence reinforces the concept that inflammatory microenvironments actively shape tumor progression.

Another major conceptual axis is the plasticity of lung cells and their microenvironment, which underlies both tumor progression and therapeutic resistance. Studies addressing hypoxia and reoxygenation (Nisar et al.) reveal how adaptive transcriptional programs promote survival, immune evasion, and epithelial–mesenchymal transition (EMT); yet, these programs remain only partially reversible upon environmental changes. Notably, this study investigates how hypoxia and post-irradiation reoxygenation influence the biological response of NSCLC cells to different radiation qualities, addressing a clinically relevant mechanism of radioresistance. Using hypoxic and reoxygenated A549 cells exposed to either X-rays or high-LET carbon ions, the authors demonstrate that hypoxia induces a persistent radioresistant phenotype following conventional photon irradiation, even after reoxygenation. In parallel, transcriptomic analyses reveal that hypoxia activates broad adaptive programs involving cell survival, EMT, inflammatory signaling, and immune evasion, many of which are substantially reversed after reoxygenation. However, this apparent, yet incomplete, transcriptional recovery following transient reoxygenation does not translate into restored radiosensitivity, indicating that hypoxia may imprint a durable resistant state that persists despite partial reversal of gene expression programs. This dissociation between transcriptional recovery and functional response suggests that additional regulatory layers, potentially including epigenetic or metabolic adaptations, may sustain resistance following hypoxic exposure.

The observation that carbon ions maintain oxygen-independent cytotoxicity further highlights their ability to overcome hypoxia-driven resistance mechanisms and supports the therapeutic potential of high-LET radiation approaches for targeting hypoxic and treatment-resistant NSCLC cell populations.

Similarly, extracellular factors and tissue-derived signals can directly modulate tumor cell behavior, as shown by the inhibitory effects of lung-derived biomolecules on proliferation and EMT-associated pathways (Samia et al.). In this study, the authors explore the capacity of soluble bioactive components derived from normal lung tissue to exert intrinsic tumor-suppressive effects in vitro. Using biomolecules extracted from lyophilized porcine freeze-dried lung tissue (FDLT), they demonstrate significant inhibition of proliferation and clonogenic growth in EGFR-mutant NSCLC cell lines, accompanied by alterations in cell-cycle progression and mitochondrial depolarization, consistent with activation of stress and cell death pathways. Importantly, FDLT treatment also suppresses EMT-associated markers, including vimentin, N-cadherin, and Twist, indicating interference with programs linked to cellular plasticity and metastatic potential.

By linking extracellular tissue-derived signals to both proliferative restraint and suppression of mesenchymal transition, this study expands the concept of microenvironmental regulation, highlighting a broader vision of tissue-intrinsic control of tumor behavior.

At the translational level, regulatory mechanisms controlling protein synthesis and signaling pathways further define disease-specific phenotypes (Reddy et al.). Importantly, this study identifies a role for the Y chromosome-linked ribosomal protein RPS4Y1 in shaping sex-specific inflammatory and remodeling responses in asthma. Using CRISPR-Cas9-dependent gene editing, the authors demonstrate that RPS4Y1-containing ribosomes may selectively regulate the translation of disease-associated mediators such as the cytokine IL6 and the extracellular matrix protein tenascin-C. Consistently, an RPS4Y1-associated transcriptional signature correlates with lung function in male asthma patients, supporting a functional link between ribosomal specialization and clinical phenotype.

From a therapeutic perspective, this Special Issue highlights diversification beyond conventional treatments, spanning targeted molecular strategies and combinatorial approaches within an expanding therapeutic landscape. Small molecules targeting oncogenic pathways, such as the PI3K/AKT axis, demonstrate selective anticancer activity and context-dependent chemosensitization in NSCLC models (Pouyfung et al.). In particular, the study characterizes a stilbene derivative as a modulator of AKT signaling, capable of reducing proliferation and migratory capacity, while exerting variable interactions with standard chemotherapy across different NSCLC cellular contexts and preferentially sparing non-tumorigenic fibroblasts, thereby indicating a degree of tumor selectivity.

Mechanistically, these effects are associated with reduced phosphorylation of AKT and its downstream effector GSK3β, without changes in total protein levels, supporting pathway-level modulation rather than protein downregulation. Notably, the compound also induces apoptosis and enhances cisplatin efficacy in a context-dependent manner, with synergistic or antagonistic interactions depending on the cellular background.

Collectively, these findings identify AKT signaling as a potential therapeutically actionable vulnerability and highlight how small-molecule interventions can elicit heterogeneous yet potentially exploitable responses across distinct NSCLC contexts, reinforcing the need for context-aware therapeutic strategies.

In parallel, advances in immunotherapy continue to reshape the clinical management of lung cancer, although challenges related to resistance, biomarker reliability, and patient stratification persist (Leone et al.). This review provides an updated and clinically oriented overview of the rapidly evolving immunotherapeutic landscape in NSCLC, with particular focus on immune checkpoint inhibitors (ICIs) and emerging combination strategies currently under clinical evaluation. By synthesizing evidence from recent and ongoing clinical trials, the authors highlight how these approaches have significantly improved outcomes in advanced disease, while also exposing persistent limitations linked to primary and acquired resistance, heterogeneous patient responses, and the limited predictive power of current biomarkers. The discussion further emphasizes the shift toward more integrated therapeutic strategies combining immunological profiling with genomic characterization, supporting a gradual transition toward more individualized treatment paradigms in NSCLC management.

These efforts are complemented by advances in diagnostic and disease-management strategies in non-oncological lung conditions (Bai J et al.). This review focuses on the evolving diagnostic landscape of rheumatoid arthritis-associated interstitial lung disease (RA-ILD), a major extra-articular complication that significantly contributes to patient mortality. The authors provide a comprehensive synthesis of recent advances, highlighting how early diagnosis remains challenging due to heterogeneous and non-specific clinical presentations. In this context, the review emphasizes the expanding role of high-resolution computed tomography (HRCT), the integration of artificial intelligence-based image analysis, and the identification of circulating and tissue-derived biomarkers as complementary tools to improve diagnostic accuracy. By aligning recent technological developments with updated clinical guidelines (released in 2025), the work outlines a more integrated and multidisciplinary diagnostic approach aimed at enabling earlier and more reliable detection of RA-ILD in clinical practice.

Importantly, this Special Issue also expands beyond cancer to include chronic and systemic lung-associated conditions, emphasizing shared molecular mechanisms across diseases. The integration of imaging, artificial intelligence, and biomarker discovery in interstitial lung disease represents a paradigm shift toward multidimensional diagnostics, combining structural and molecular information.

Likewise, the modulation of airway epithelial functions, including mucin production and inflammatory signaling, provides insights into the molecular basis of respiratory disorders such as asthma (Yoshio et al.). This study specifically examines the ability of liquiritin, a flavonoid compound, to modulate airway mucus dysregulation in asthma-relevant epithelial cells. Using the NCI-H292 human airway epithelial model, the authors demonstrate that liquiritin reduces the production and secretion of MUC5AC and MUC5B in a dose-dependent manner, counteracting basal mucin overproduction. Notably, these effects are maintained under conditions of phorbol 12-myristate 13-acetate (PMA)-induced stimulation, a well-established model of excessive mucin secretion, where liquiritin significantly attenuates the induced upregulation of both mucins. Mechanistically, these effects are associated with suppression of ERK and p38 MAPK signaling pathways, further confirmed under PMA challenge. These findings link epithelial secretory dysfunction to inflammatory signaling cascades, highlighting mucin regulation as a modifiable component of airway disease pathology.

Collectively, the studies presented here converge on a unifying concept: lung diseases are governed by interconnected molecular networks that transcend traditional diagnostic categories. Immune signaling, metabolic adaptation, tissue remodeling, and systemic communication dynamically shape disease initiation, progression, and therapeutic response, underscoring the need to move beyond descriptive pathology toward predictive and personalized medicine. Rather than reiterating biological integration per se, this Special Issue explores shared molecular convergences across distinct lung diseases while preserving context-dependent differences that define disease specificity and clinical heterogeneity, reflecting a broader scientific perspective.

Building on these advances, we have launched a second edition of this Special Issue to further investigate emerging molecular mechanisms and therapeutic strategies. This continued effort aims to define conserved regulatory principles alongside disease-specific adaptations, supporting a convergence-oriented yet context-aware framework for lung disease research.

Together, these studies provide a conceptual and translational foundation for future research and therapeutic development in pulmonary diseases, moving decisively beyond traditional disciplinary boundaries.

## Figures and Tables

**Figure 1:**
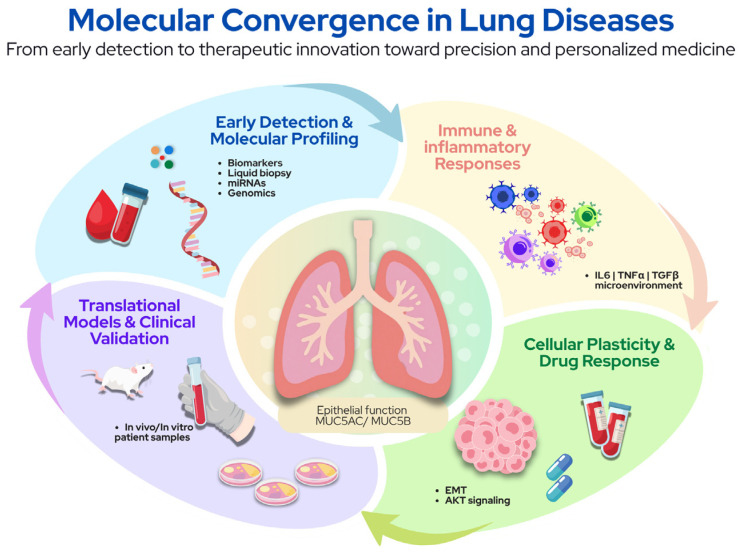
Molecular convergence across lung diseases. Schematic representation of the interconnected molecular processes underlying lung diseases, integrating early detection strategies, immune and inflammatory responses, cellular plasticity, and translational validation. Together, these converging axes support the development of precision and personalized therapeutic approaches.

**Table 1:** List of the articles present in this Special Issue.

First Author	Reference
Morjani, O	Morjani et al. Int. J. Mol. Sci. 2025, 26, 11014. https://doi.org/10.3390/ijms262211014
Zedan, Y	Zedan et al. Int. J. Mol. Sci. 2025, 26, 12035. https://doi.org/10.3390/ijms262412035
Savin, I.A	Savin et al. Int. J. Mol. Sci. 2026, 27, 273. https://doi.org/10.3390/ijms27010273
Bai, H	Bai et al. Int. J. Mol. Sci. 2025, 26, 2549. https://doi.org/10.3390/ijms26062549
Sanchez-Rojas, M.J	Sanchez-Rojas et al. Int. J. Mol. Sci. 2025, 26, 5073. https://doi.org/10.3390/ijms26115073
Nisar, H	Nisar et al. Int. J. Mol. Sci. 2025, 26, 9153. https://doi.org/10.3390/ijms26189153
Samia, U	Samia et al. Int. J. Mol. Sci. 2025, 26, 11743. https://doi.org/10.3390/ijms262311743
Reddy, K.D	Reddy et al. Int. J. Mol. Sci. 2025, 26, 6213. https://doi.org/10.3390/ijms26136213
Pouyfung, P	Pouyfung et al. Int. J. Mol. Sci. 2026, 27, 719. https://doi.org/10.3390/ijms27020719
Leone, G.M	Leone et al. Int. J. Mol. Sci. 2025, 26, 11055. https://doi.org/10.3390/ijms262211055
Bai, J	Bai et al. Int. J. Mol. Sci. 2026, 27, 1165. https://doi.org/10.3390/ijms27031165
Yoshio, R	Yoshio et al. Int. J. Mol. Sci. 2025, 26, 8076. https://doi.org/10.3390/ijms26168076

